# Molecular analysis of *Mycobacterium *isolates from extrapulmonary specimens obtained from patients in Mexico

**DOI:** 10.1186/1472-6890-9-1

**Published:** 2009-03-09

**Authors:** Cosme Alvarado-Esquivel, Nora García-Corral, David Carrero-Dominguez, José Antonio Enciso-Moreno, Teodoro Gurrola-Morales, Leopoldo Portillo-Gómez, Rudi Rossau, Wouter Mijs

**Affiliations:** 1 Department of Microbiology, Faculty of Medicine, Juárez University of Durango State, Durango, Mexico; 2 Department of Pharmacology, Faculty of Medicine, Juárez University of Durango State, Durango, Mexico; 3 Regional Laboratory of Epidemiological Reference, Mexican Social Security Institute, Guadalajara, Mexico; 4 Medical Research Unit, Mexican Social Security Institute, Zacatecas, México; 5 Department of Pathology, Faculty of Medicine, Juárez University of Durango State, Durango, Mexico; 6 Department of Microbiology and Parasitology, University Center of Health Sciences, University of Guadalajara, Guadalajara, Mexico; 7 R&D Diagnostics, Innogenetics NV, Gent, Belgium

## Abstract

**Background:**

Little information is available on the molecular epidemiology in Mexico of *Mycobacterium *species infecting extrapulmonary sites in humans. This study used molecular methods to determine the *Mycobacterium *species present in tissues and body fluids in specimens obtained from patients in Mexico with extrapulmonary disease.

**Methods:**

Bacterial or tissue specimens from patients with clinical or histological diagnosis of extrapulmonary tuberculosis were studied. DNA extracts from 30 bacterial cultures grown in Löwenstein Jensen medium and 42 paraffin-embedded tissues were prepared. Bacteria were cultured from urine, cerebrospinal fluid, pericardial fluid, gastric aspirate, or synovial fluid samples. Tissues samples were from lymph nodes, skin, brain, vagina, and peritoneum. The DNA extracts were analyzed by PCR and by line probe assay (INNO-LiPA MYCOBACTERIA v2. Innogenetics NV, Gent, Belgium) in order to identify the *Mycobacterium *species present. DNA samples positive for *M. tuberculosis *complex were further analyzed by PCR and line probe assay (INNO-LiPA Rif.TB, Innogenetics NV, Gent, Belgium) to detect mutations in the *rpo*B gene associated with rifampicin resistance.

**Results:**

Of the 72 DNA extracts, 26 (36.1%) and 23 (31.9%) tested positive for *Mycobacterium species *by PCR or line probe assay, respectively. In tissues, *M. tuberculosis *complex and *M. genus *were found in lymph nodes, and *M. genus *was found in brain and vagina specimens. In body fluids, *M. tuberculosis *complex was found in synovial fluid. *M. gordonae*, *M. smegmatis*, *M. kansasii*, *M. genus*, *M. fortuitum/M. peregrinum *complex and *M. tuberculosis *complex were found in urine. *M. chelonae/M. abscessus *was found in pericardial fluid and *M. kansasii *was found in gastric aspirate. Two of *M. tuberculosis *complex isolates were also PCR and LiPA positive for the *rpo*B gene. These two isolates were from lymph nodes and were sensitive to rifampicin.

**Conclusion:**

1) We describe the *Mycobacterium *species diversity in specimens derived from extrapulmonary sites in symptomatic patients in Mexico; 2) Nontuberculous mycobacteria were found in a considerable number of patients; 3) Genotypic rifampicin resistance in *M. tuberculosis *complex infections in lymph nodes was not found.

## Background

The genus *Mycobacterium *has been classified into many species [1]. A group of *Mycobacterium *species called *M. tuberculosis *complex that comprise *M. tuberculosis*, *M. bovis*, *M. microti*, and *M. africanum *is of utmost clinical importance since it causes tuberculosis in humans worldwide [2-4]. *Mycobacterium *species other than those of the tuberculosis complex, also called nontuberculous mycobacteria, are widely distributed in the environment and may colonize and occasionally cause infections in humans [5-7]. Mycobacteria of the *M. tuberculosis *complex and nontuberculous mycobacteria have been found to cause infections in immunocompetent and immunocompromised subjects and cause pathology in pulmonary and extrapulmonary sites [8-11]. Most epidemiological studies on mycobacteria have been focused on pulmonary infections, while extrapulmonary infections have been poorly explored. Extrapulmonary tuberculosis accounts for about 10% to 20% of tuberculosis cases in immunocompetent subjects but this frequency increases markedly in immunocompromised subjects [12]. Extrapulmonary sites are affected in up to 60% of patients suffering from acquired immunodeficiency syndrome and tuberculosis [12]. Molecular diagnosis of mycobacterial infections has enabled rapid detection of species in clinical specimens, detection of drug resistance, and typing for epidemiological studies [4,6,9,13]. Little is known on the worldwide molecular epidemiology of *Mycobacterium *species infecting extrapulmonary sites. In addition, genotypic resistance to drugs in *M. tuberculosis *isolates from extrapulmonary sites has been poorly studied. There is scarce information on the molecular epidemiology in Mexico of *Mycobacterium *species infecting extrapulmonary sites. Therefore, in this study on samples obtained from such patients, we used molecular methods to identify the *Mycobacterium *species in tissue samples and body fluids. In addition, we analyzed the gene encoding for the β-subunit of the RNA polymerase (*rpo*B) for identification of mutations associated with rifampicin resistance in DNA extracts positive for *M. tuberculosis *complex.

## Methods

### Patients and specimens

Seventy-two patients with clinical or histological findings compatible with extrapulmonary tuberculosis were studied. Patients attended four public hospitals in the Mexican cities of Durango, Zacatecas and Guadalajara. Durango City is located in north central Mexico, while Zacatecas and Guadalajara Cities are located in central Mexico. Specimens from the patients were prepared as either paraffin-embedded tissue or cultures of body fluids. For paraffin-embedded tissues, we studied recent and stored specimens obtained from the pathology departments of two hospitals from 2002 to 2008. In total, tissues of 42 patients were studied: twelve of them were obtained in Durango City and 30 in Zacatecas City. For body fluids, we studied recent and stored cultures in Löwenstein Jensen medium from urine, pericardial fluid, gastric aspirate, synovial fluid and cerebrospinal fluid obtained from 30 patients. These specimens were obtained in two hospitals in Guadalajara City from 2007 to 2008. The studied specimens were obtained only from the participating hospitals and are not representative of specimens from all hospitals in these cities. DNA was extracted from all specimens using a commercially available kit (QIAamp DNA Mini Kit, QIAGEN. Germany) following the instructions of the manufacturer. Prior to extraction, paraffin-embedded tissues were treated with xylene and alcohol to remove the paraffin, and bacterial cultures were heated at 95°C for 10 minutes.

### Molecular analysis of *Mycobacterium *species

DNA extracted from the 72 specimens was amplified by PCR using the INNO-LiPA MYCOBACTERIA v2 Amp. (Innogenetics NV, Gent, Belgium) kit according to the manufacturers' instructions. PCR parameters were: denaturation at 95°C for 1 min followed by 40 cycles of denaturation at 95°C for 30 sec, annealing at 62°C for 30 sec, and extension at 72°C for 30 sec. Electrophoresis of amplified products was performed in a 2% agarose gel. The gel was then stained with ethidium bromide and visualized by ultraviolet transillumination. In addition, amplified products were analyzed by line probe assay (INNO-LiPA MYCOBACTERIA v2. Innogenetics NV, Gent, Belgium) following the manufacturers' instructions. This DNA probe test targets the 16S–23S ribosomal RNA spacer region and detects and identifies the genus *Mycobacterium *and 16 different mycobacterial species. As a quality control for amplifications, we included water samples as negative controls, and *M. tuberculosis *complex DNA as positive controls in each run.

### Analysis of rifampicin resistance of *M. tuberculosis*

Amplification of the rifampicin resistance region of the gene encoding for the β-subunit of the RNA polymerase (*rpo*B) in DNA samples positive for *M. tuberculosis *complex by INNO-LiPA MYCOBACTERIA v2 was performed using the INNO-LiPA Rif.TB Amplification kit (Innogenetics NV, Gent, Belgium). The PCR parameters were denaturation at 95°C for 5 min followed by 30 cycles of denaturation at 95°C for 1 min, annealing at 55°C for 1 min, extension at 72°C for 1 min, and an elongation at 72°C for 10 min. After electrophoresis of the amplified products in 2% agarose gel, the gel was stained with ethidium bromide and visualized by ultraviolet transillumination. In addition, amplification products were analyzed by line probe assay (INNO-LiPA Rif.TB. Innogenetics NV, Gent, Belgium) for detection of rifampicin resistance. This assay was performed following the manufacturers' instructions. As a quality control for amplifications, we included water samples as negative controls, and *M. tuberculosis *complex DNA as positive controls in each run.

## Results and discussion

### Rate of positive PCR and LiPA samples

As shown in Tables 1 and 2, only 26 (36.1%) and 23 (31.9%) of the 72 DNA extracts were PCR positive for the 16S–23S ribosomal RNA spacer region of *Mycobacterium *species and LiPA hybridization, respectively. The low rate of positive samples could be explained by: 1) No *Mycobacterium *species were present in the samples: pathology diagnosis is not conclusive of tuberculosis; 2) DNA of *Mycobacterium *was present in the tissues but at levels that were undetectable by a single-round PCR assay; 3) DNA preservation in some samples was sub-optimal; and 4) the presence of PCR inhibitors. Most samples tested were archival and a comparable rate of positive PCR results in archival tissue samples was reported in a previous study [14]. Moreover, our study was based in a single determination and it is possible that repeating assays could increase the rate of positive samples.

**Table 1:** Clinical and histological data of paraffin embedded tissues from the study population.

Case No.	Age	Gender	Place of origin	Clinical diagnosis	Specimen	Pathology diagnosis	ZN staining	PCR	Mycobacteria
T1	39	M	Dgo	TB	Brain	TB	+	+	*M. genus*
T2	34	M	Dgo	Tumour	Lymph node	TB	+	+	*M. tuberculosis *complex
T3	42	M	Dgo	Lymphadenitis	Lymph node	TB	-	-	
T4	45	F	Dgo	Tumour	Vagina	TB	+	+	*M. genus*
T5	45	M	Dgo	Lymphadenitis	Lymph node	TB	+	+	*M. genus*
T6	18	M	Dgo	Tumour	Lymph node	TB	+	-	
T7	40	M	Dgo	TB	Lymph node	TB	-	-	
T8	13	M	Dgo	Acute abdomen	Epiplon	TB	+	-	
T10	21	F	Dgo	Lymphadenitis	Lymph node	TB	-	-	
T11	29	M	Dgo	Lymphadenitis	Lymph node	TB	ND	-	
T12	16	M	Dgo	Acute abdomen	Peritoneum	TB	-	-	
T14	20	M	Dgo	Epidermic cyst	Skin	TB	+	-	
T41	9	F	Zac	Granuloma	Lymph node	TB	+	+	*M. tuberculosis *complex
T42	57	M	Zac	Granuloma	Lymph node	TB	ND	-	
T43	31	M	Zac	TB	Lymph node	TB	+	-	*M. tuberculosis *complex
T44	56	M	Zac	Granuloma	Lymph node	TB	ND	-	*M. genus*
T45	1	M	Zac	Granuloma	Lymph node	TB	ND	-	*M. genus*
T46	39	M	Zac	Granuloma	Lymph node	TB	+	-	
T47	39	M	Zac	Granuloma	Lymph node	TB	+	-	
T48	1	M	Zac	Granuloma	Lymph node	TB	+	-	
T49	53	M	Zac	Lymphoma	Lymph node	Lymphoma	ND	-	
T50	1	F	Zac	Granuloma	Lymph node	TB	ND	-	
T51	1	M	Zac	Granuloma	Lymph node	TB	ND	-	
T52	62	F	Zac	Hyperplasia	Lymph node	Hyperplasia	ND	-	
T53	19	M	Zac	Lymphoma	Lymph node	TB	ND	-	
T54	67	F	Zac	Lymphoma	Lymph node	Lymphoma	ND	-	*M. tuberculosis *complex
T55	37	F	Zac	Hyperplasia	Lymph node	Hyperplasia	ND	-	*M. tuberculosis *complex
T56	4	M	Zac	Granuloma	Lymph node	Lymphoma	ND	-	
T57	44	F	Zac	Granuloma	Lymph node	TB	+	-	
T58	32	F	Zac	Granuloma	Lymph node	TB	ND	-	
T59	32	F	Zac	Granuloma	Lymph node	TB	+	-	
T60	74	F	Zac	Granuloma	Lymph node	TB	+	-	
T61	38	M	Zac	Granuloma	Lymph node	TB	+	-	
T62	9	M	Zac	Granuloma	Lymph node	TB	ND	-	
T63	0.4	M	Zac	Granuloma	Lymph node	TB	ND	+	
T64	53	F	Zac	Granuloma	Lymph node	TB	+	-	
T65	45	F	Zac	Granuloma	Lymph node	TB	ND	-	
T66	1	M	Zac	Granuloma	Lymph node	TB	+	-	
T67	3	M	Zac	Granuloma	Lymph node	TB	ND	-	
T68	54	F	Zac	Granuloma	Lymph node	TB	ND	-	
T69	72	F	Zac	Lymphoma	Lymph node	Lymphoma	ND	-	
T70	28	F	Zac	Granuloma	Lymph node	TB	ND	-	

ND: Not done. Dgo: Durango. Zac: Zacatecas.

**Table 2:** Clinical data of patients with Löwenstein Jensen cultures strains.

Case No.	Age	Gender	Clinical diagnosis	Specimen	ZN staining	PCR	Mycobacteria
C1	65	M	RTB	Urine	-	-	
C2	10	M	RTB	Urine	+	+	
C3	39	F	RTB	Urine	+	+	*M. gordonae*
C4	29	M	RTB	Urine	ND	+	
C5	48	F	RTB	Urine	+	-	
C6	29	F	RTB	Urine	+	-	
C7	12	M	RTB	Urine	+	-	
C8	23	F	RTB	Urine	-	-	
C9	79	F	RTB	Urine	+	-	
C10	23	F	RTB	Urine	ND	+	
C11	20	M	TB	Pericardial fluid	ND	+	*M. chelonae *complex group III. *M. abscessus*
C12	29	F	RTB	Urine	ND	+	*M. tuberculosis *complex
C13	38	F	RTB	Urine	-	+	*M. smegmatis*
C14	53	F	RTB	Urine	-	+	*M. smegmatis*
C15	63	F	RTB	Urine	+	-	*M. smegmatis*
C16	13	F	RTB	Urine	ND	+	*M. kansasii *group II
C17	3	F	RTB	Urine	ND	+	*M. genus*
C18	35	F	TB	Gastric aspirate	ND	+	*M. kansasii *group I
C19	9	F	RTB	Urine	ND	+	
C20	13	F	RTB	Urine	ND	+	*M. fortuitum-M. peregrinum *complex
C21	48	F	RTB	Urine	+	+	*M. smegmatis*
C22	34	F	RTB	Urine	ND	+	*M. genus*
C23	23	F	RTB	Urine	ND	+	
C24	29	M	RTB	Urine	ND	+	
C25	35	M	RTB	Urine	ND	+	
C26	10	M	RTB	Urine	+	-	
C27	1	F	TB	CSF	+	-	
C28	30	F	TB	Synovial fluid	+	+	*M. tuberculosis *complex
C29	45	M	TB	CSF	-	-	
C30	56	F	RTB	Urine	+	+	

CSF: Cerebrospinal fluid

RTB: Renal tuberculosis

### *Mycobacterium *species

Table 1 shows the *Mycobacterium *species identified in paraffin-embedded tissues, and Table 2 shows results obtained for body fluids. In paraffin-embedded tissues, *M. tuberculosis *complex and *M. genus *were found in 5 and 3 lymph nodes, respectively. The predominant *M. tuberculosis *complex infection in lymph nodes found in our patients is consistent with results in patients with lymphadenitis reported by other researchers [4,15,16]. With respect to other tissues, *M. genus *was found in specimens of brain and vagina. Hybridization with *M. genus *probes indicates that amplified DNA belongs to mycobacteria but it is not one of the 16 *Mycobacterium *species included in the kit. In body fluids, *M. tuberculosis *complex and nontuberculous mycobacteria were found in 1 (10%) and 9 (90%) of urine samples with positive hybridization, respectively. The frequency of *M. tuberculosis *complex infection in these patients is lower than that reported in a study performed in Turkey, where 12 (70.5%) of 17 strains isolated from urine cultures of patients with suspected urinary tuberculosis were identified as *M. tuberculosis *complex [17]. The finding of a high frequency of nontuberculous mycobacteria in urine may indicate contamination by mycobacteria in the environment. Nevertheless, all urine samples were obtained from symptomatic subjects with clinical diagnosis of renal tuberculosis and had obtained clinical improvement after anti-tuberculosis therapy. Many nontuberculous mycobacteria have recently been recognized as pathogenic [18]. The species of nontuberculous mycobacteria found in the urine of our patients have been found in symptomatic patients in several studies: 1) *M. fortuitum *was found in 2 of 5 renal transplant recipients with urinary symptoms [19]; 2) a case report of a woman suffering from urinary complaints describes that *M. tuberculosis *and other pathogens were not detected, but that *M. gordonae *was repeatedly isolated from urine. The patient responded to a standard anti-tuberculosis regimen [20]; and 3) positive urine culture for *M. kansasii *in patients with persistent fever who were suffering from hairy cell leukemia and disseminated atypical mycobacterial infection [21]. On the other hand, *M. smegmatis *has been isolated from skin and soft tissue infections [22], but to the best of our knowledge there is not any report of its isolation in urine from patients with urinary disease. The finding of nontuberculous mycobacteria in any specimen should always be correlated with clinical data of the patients to make appropriated therapeutic decisions. Since most *Mycobacteriu*m species found in extrapulmonary specimens in our symptomatic patients did not belong to the *M. tuberculosis *complex, our results emphasize the importance of performing identification of mycobacterial species. Discrimination of infecting *Mycobacterium *species is important since treatment differs [23]. PCR and LiPA technology has been successfully used in identifying both *Mycobacterium *species and rifampicin resistance in *M. tuberculosis *isolates in either *Mycobacterium *grown in culture or directly in some clinical samples [24-27]. However, this study is the first to identify and classify *Mycobacterium *species and to detect genotypic rifampicin resistance from paraffin-embedded tissues by using LiPA technology. In addition, this is the first report of *Mycobacterium *species diversity detected by molecular methods in symptomatic patients in Mexico suffering from extrapulmonary disease. Our results indicate that LiPA can be used successfully in analyzing DNA extracts of paraffin-embedded tissues.

### Rifampicin resistance

Two of the seven *M. tuberculosis *complex samples were positive for amplification of the *rpo*B gene and showed hybridization patterns compatible with wild type *M. tuberculosis*. Samples negative for LiPA were not further analyzed. The two positive samples were obtained from lymph nodes and were considered sensitive to rifampicin. These results further provide molecular characterization of the *M. tuberculosis *complex isolates. Patients suffering from extrapulmonary tuberculosis have a delay in diagnosis [28]. Therefore, fast molecular analysis of *Mycobacterium *using LiPA technology provides an aid for rapid diagnosis and the opportunity to take optimal preventive and treatment measures.

## Conclusion

1) We describe the *Mycobacterium *species diversity in specimens derived from extrapulmonary sites in symptomatic patients in Mexico; 2) Nontuberculous mycobacteria were found in a considerable number of patients; 3) genotypic rifampicin resistance in *M. tuberculosis *complex infections in lymph nodes was not found.

## Competing interests

The authors declare that they have no competing interests.

## Authors' contributions

CAE conceived and designed the study protocol, participated in the coordination and management of the study, performed laboratory tests and data analysis, and wrote the manuscript. NGC performed the laboratory tests and data analysis. LPG and DCD performed *Mycobacteria *cultures and data analysis. TGM performed the histological evaluation of paraffin-embedded tissues. JAEM prepared DNA extracts from tissues and performed data analysis. RR and WM performed data analysis and wrote the manuscript.

## Pre-publication history

The pre-publication history for this paper can be accessed here:


